# *De novo* Taproot Transcriptome Sequencing and Analysis of Major Genes Involved in Sucrose Metabolism in Radish (*Raphanus sativus* L.)

**DOI:** 10.3389/fpls.2016.00585

**Published:** 2016-05-17

**Authors:** Rugang Yu, Liang Xu, Wei Zhang, Yan Wang, Xiaobo Luo, Ronghua Wang, Xianwen Zhu, Yang Xie, Benard Karanja, Liwang Liu

**Affiliations:** ^1^National Key Laboratory of Crop Genetics and Germplasm Enhancement, College of Horticulture, Nanjing Agricultural UniversityNanjing, China; ^2^School of Life Science, Huaibei Normal UniversityHuaibei, China; ^3^Department of Plant Sciences, North Dakota State UniversityFargo, ND, USA

**Keywords:** *Raphanus sativus*, taproot, RNA-seq, transcriptome, sucrose metabolism

## Abstract

Radish (*Raphanus sativus* L.) is an important annual or biennial root vegetable crop. The fleshy taproot comprises the main edible portion of the plant with high nutrition and medical value. Molecular biology study of radish begun rather later, and lacks sufficient transcriptomic and genomic data in pubic databases for understanding of the molecular mechanism during the radish taproot formation. To develop a comprehensive overview of the ‘NAU-YH’ root transcriptome, a cDNA library, prepared from three equally mixed RNA of taproots at different developmental stages including pre-cortex splitting stage, cortex splitting stage, and expanding stage was sequenced using high-throughput Illumina RNA sequencing. From approximately 51 million clean reads, a total of 70,168 unigenes with a total length of 50.28 Mb, an average length of 717 bp and a N50 of 994 bp were obtained. In total, 63,991 (about 91.20% of the assembled unigenes) unigenes were successfully annotated in five public databases including NR, GO, COG, KEGG, and Nt. GO analysis revealed that the majority of these unigenes were predominately involved in basic physiological and metabolic processes, catalytic, binding, and cellular process. In addition, a total of 103 unigenes encoding eight enzymes involved in the sucrose metabolism related pathways were also identified by KEGG pathway analysis. Sucrose synthase (29 unigenes), invertase (17 unigenes), sucrose-phosphate synthase (16 unigenes), fructokinase (17 unigenes), and hexokinase (11 unigenes) ranked top five in these eight key enzymes. From which, two genes (*RsSuSy1, RsSPS1*) were validated by T-A cloning and sequenced, while the expression of six unigenes were profiled with RT-qPCR analysis. These results would be served as an important public reference platform to identify the related key genes during taproot thickening and facilitate the dissection of molecular mechanisms underlying taproot formation in radish.

## Introduction

Radish (*Raphanus sativus* L., 2*n* = 2*x* = 18) is an important root vegetable crop belonging to the Brassicaceae family grown all over the world, especially in East Asia (Johnston et al., 2005; Wang and He, 2005). The fleshy taproot is a key organ for the direct yield and quality of radish, and its formation and development is a complex biological processes involving morphogenesis and dry matter accumulation (Wang and He, 2005). During this process, an abundance of storage compounds are synthesized, including carbohydrates, ascorbic acid, folic acid, potassium, vitamin B6, riboflavin, magnesium and sulforaphane, which mainly could determine the economic value of radish taproot and provide nutrients and medicinal function for human beings (Curtis, 2003; Gutiérrez and Perez, 2004; Chaturvedi, 2008; Wang et al., 2013). Hence, understanding the processes regulating the root formation and development is of particular importance.

To date, large amounts of transcriptomic and genomic sequences have been provided in model plants, such as Arabidopsis, Antirrhinum and rice, which have greatly helped the understanding of the complexity of growth and development in higher plants. For radish, many researches reported that the genomic information have recently been analyzed. In recent years, the genomic and transcriptome information of radish have been extensively clarified. For instance, two leaf and two root transcriptomes from radish were reported (Wang et al., 2012, 2013; Zhang et al., 2013; Wu et al., 2015), and some critical genes associated with glucosinolate metabolism and heavy metal stress response were identified (Wang et al., 2013). In addition, 314,823 expressed sequence tags (ESTs), 31,935 nucleotide sequences and 16 genome survey sequences (GSS) were stored in NCBI for radish (http://www.ncbi.nlm.nih.gov/nucest/?term=radish) (March 7th, 2014). More recently, the draft genome sequences of *R. sativus* have been assembled and published (Kitashiba et al., 2014). These data might provide the useful database for genomic and functional investigation on some important horticultural traits in radish. However, the formation and development of taproot is a complex biological process in radish. The radish advanced inbred line, ‘NAU-YH’ with a taproot in very small size (maximum diameter <3.0 cm at maturity), is a very suitable genotype for taproot development investigation. However, the genome and the transcriptome of ‘NAU-YH’ were not sequenced, and the related genomic information was still unavailable. The resulting data of transcriptome sequencing of this genotype would be useful for further molecular investigation on taproot development.

Sucrose is the major product of photosynthesis (Ruan, 2012). Generally, it is the main form of assimilated carbon to be transported from “source” to “sink” organs in higher plants (Farrar et al., 2000). In addition, sucrose is not only the source of carbon skeletons which may involve in the synthesis essential metabolite compounds including starch, cellulose and proteins (Weber et al., 1997; Cheng and Chourey, 1999; Babb and Haigler, 2001), but also an important signal molecule in plants that regulates the expression of microRNA (Yang et al., 2013), transcription factors (Xiong et al., 2013), plant hormone (Stokes et al., 2013), and other genes (Ruan, 2014). Therefore, sucrose metabolism plays important roles in plant growth and development.

There are three key enzymes responsible for sucrose synthesis and degradation in plants, including invertase (INV, EC 3.2.1.26, involved in sucrose degradation), sucrose synthase (SuSy, EC 2.4.1.13, involved in sucrose degradation), and sucrose-phosphate synthase (SPS, EC 2.3.1.14, involved in sucrose synthesis; Ren and Zhang, 2013). During last decade, extensive knowledge of sucrose metabolism has been studied by cloning and characterizing the genes encoding key enzymes in various plant species. For example, *SuSy* gene activity was found to be related to sink energy in tomato fruit (Wang et al., 1993); The gene structure, expression and regulation, and the physiological functions of the key enzymes involved in sucrose metabolism in maize were reviewed by Ren and Zhang (2013); Li and Zhang (2003) reported that SuSy was the most actively expressed enzyme in sucrose metabolism in developing storage root and was correlated with sink strength, while invertase was active at cell formation stages in Sweet Potato. The fleshy taproot of radish is one of major sink organ. Its growth and development requires an increase in sink activity, which is obtained by activating sucrose metabolism. Usuda et al. (1999) found that sink activity was strongly related to the level and activity of sucrose synthase but not to the activity of invertase. Wang et al. (2007) also found that the activities of SuSy were similar to sink activities in all lines. These results suggested that these enzymes might be associated with the developmental of the sink organ of radish. To date, although several studies have reported the role of sucrose metabolism in radish taproot thickening growth (Rouhier and Usuda, 2001; Wang et al., 2007), molecular mechanisms underlying sucrose metabolism remains unclear, especially for identification and evaluation of the full range of gene involved in sucrose metabolism in radish taproot.

Next-generation sequencing (NGS)-based RNA sequencing for transcriptome methods (RNA-seq) has been proven to be an effective method to analyze functional gene variation, and dramatically improve the speed and efficiency of gene discovery. (Angeloni et al., 2011; Hyun et al., 2012; Ward et al., 2012). The aim of this study was to obtain a comprehensive survey of transcripts associated with radish taproot formation. We utilize Illumina paired-end Solexa sequencing to conduct the *de novo* assembly and annotation of the ‘NAU-YH’ taproot transcriptome. According to KEGG pathway information, we first identified candidate genes of the key enzymes involved in sucrose metabolism and estimated the expression levels of these genes in different stages of taproot thickening. These results would provide important information for identifying the related key genes during taproot formation and facilitate further understanding of molecular mechanisms underlying taproot thickening in radish.

## Materials and methods

### Plant material and RNA extraction

The radish (*R. sativus* L.) advanced inbred line ‘NAU-YH’ was chosen for this study. Seeds were selected and germinated on moist filter paper in darkness for 3 days. Then, seedlings were transplanted into plastic pots containing 1:1 mixture of soil and peat substrate, and cultured in the greenhouse at Nanjing Agricultural University. The development of cortex splitting is an important signal of the initiation of thickening growth of taproot in radish due to the cortex cells cannot divide and expand (Wang and He, 2005). Moreover, according to the ‘NAU-YH’ radish established morphological traits, the root cortex split initiated about 12 days after sowing (DAS), and the full root cortex splitting was achieved over a period of 22 DAS. The growth of root indicated rapidly thickening in the 22 to 42 DAS, then continued into a slowly thickening period. Therefore, samples of taproots were collected at three different development stages: 10 (DAS) (Stage 1, pre-cortex splitting stage), 20 DAS (Stage 2, cortex splitting stage), and 40 DAS (Stage 3, expanding stage) in this study. The subsamples of taproot, stem and leaf tissues were collected at 10, 20, 40, and 50 DAS, respectively for RT-qPCR verification. All samples were frozen in liquid nitrogen and stored at –80°C for further use.

Total RNA was extracted separately from the three taproot samples using Trizol regent (Invitrogen, USA) following the manufacturer's protocol. After the RNase-free DNase I (Takara, Japan) treatment, for cDNA preparation, a total 20 μg of RNA was mixed equally from each of the three taproot samples.

### cDNA library construction and sequencing

After the total RNA extraction, mRNA was purified from the 20 μg of RNA using Sera-mag Magnetic Oligo (dT) Beads (Thermo Fisher Scientific, USA). Then the purified mRNA was broken into small pieces using fragmentation buffer under elevated temperature. These short fragments as templates were used to synthesize first strand cDNA. Subsequently, the second-strand cDNA was synthesized using the SuperScript Double-Stranded cDNA Synthesis Kit (Invitrogen, USA). The short cDNA fragments were purified with Qia-Quick PCR extraction kit and end-repair with EB buffer and ligation of A-tailing. Next, suitable fragments were selected as templates for PCR amplification to create the final cDNA library. Finally, after validating on an Agilent Technologie 2100 Bioanalyzer and ABI StepOnePlus Real-Time PCR System, the cDNA library was sequenced at the Beijing Genomics Institute (BGI, Shenzhen, China) using Illumina HiSeq™ 2000 sequencing platform. Image data outputs from sequencing machine were transformed by base calling into sequence data, which is called raw reads.

### Data filtering and *De novo* assembly

The clean reads were generated by removing adaptor reads, empty reads, and low quality reads from the raw reads. Then, the clean reads were assembled using a *de novo* assembly program Trinity (Grabherr et al., 2011) with default K-mers = 25. Briefly, the process was done as previously described procedure (Wang et al., 2013). The clean reads with a certain length of overlap were firstly used to produce contigs. The reads were then mapped back to the contigs, and the paired-end reads was used to detect contigs from the same transcript as well as the distances between these contigs. To reduce any sequence redundancy, the contigs were further connected using Trinity after the paired-end reads, and sequences that could not be extended on either end were defined as unigenes. Finally, the unigenes were divided into two classes by gene family clustering. One is clusters, several unigenes with over 70% similarity between them, and the other unigenes were singletons.

### Functional annotation and classification

The assembled unigene sequences were aligned by BLASTx to the publicly available protein databases which included NCBI non redundant protein (Nr), Gene Ontology (GO), Clusters of Orthologous Groups (COG), Swiss-Prot protein and the Kyoto Encyclopedia of Genes and Genomes (KEGG), and aligned by BLASTn to nucleotide databases (Nt) with an *E* ≤ 10^−5^. The best alignments in blast results were taken to decide the coding region sequences of the assembled unigenes. If the results from different databases conflicted with each other, a priority order of Nr, Swiss-Prot, KEGG and COG was followed. Meanwhile, if the assembled unigene sequences could not be aligned to any database, the software ESTScan was used to predict the protein coding sequence (CDS) and its sequence orientation (Iseli et al., 1999). And then, GO annotation of the unigenes was performed based on the best hits from Nr annotation using BLAST2GO program (Conesa et al., 2005), and the results of GO annotation were further used to conduct GO functional classification by WEGO software (Ye et al., 2006).

### Gene validation by T-A cloning and sequencing

According to the conserved region of radish EST sequences from radish cDNA library, the specific PCR primers of the two selected genes were designed to isolate sucrose metabolism related genes using Primer 5.0 software (Table S1). PCR was performed according to the method described previously (Wang et al., 2013). The PCR products were separated and ligated into the pMD18-T vector (Takara Bio Inc., China), and then transformed into *E. coli* DH5α. Positive clones were sequenced with ABI 3730 (Applied Bio systems, USA).

### RT-qPCR analysis

Six selected unigenes with crucial roles in sucrose metabolism were selected for RT-qPCR analysis using the SYBR Green Master ROX (Roche, Japan). The unigenes specific primers were designed using Beacon Designer 7.0 software (Table S1). Total RNAs were respectively extracted from taproot, stems and leaves in four different taproot development stages (10, 20, 40, and 50 DAS) using Trizol® Reagent (Invitrogen, USA) and then treated with PrimeScript® RT reagent Kit (Takara, Dalian, China) to reverse transcribe into cDNA. The amplification reactions were run in iCycler iQ real-time PCR detection system (BIO-RAD) according to previous reports (Xu et al., 2012). All reactions were performed in three replicates and the equation ratio = 2${}^{-\Delta \Delta {\text{C}}_{T}}$ was applied to calculate the relative expression level of the selected unigenes using *Actin* gene as the internal control gene. The data were analyzed using the Bio-Rad CFX Manager software.

## Results and discussion

### Sequencing and *De novo* transcriptome assembly

To obtain an overview of ‘NAU-YH’ transcriptome in taproots, and identify candidate genes involved in sucrose metabolism, a cDNA library was constructed from the RNA (an equally mixture of total RNA from three taproot developmental stages) of ‘NAU-YH’, and sequenced using the Illumina HiSeq™ 2000 sequencing platform. The Illumina sequencing results were shown in Table 1. It yielded a total of 57.0 million raw sequencing reads. After the adapter sequences, reads with unknown nucleotides larger than 5% and low quality reads were removed, 51.1 million clean pair-end reads with total of 4.6 billion nucleotides (nt) were generated for assembly. Q20 percentage, N percentage, and GC percentage were 98.29, 0.01, and 47.10%, respectively. The output was similar to previous studies on radish taproot transcriptome (Wang et al., 2012, 2013). In addition, the length of assembled sequences is an evaluation criterion for the assembly of transcriptome. In this study, the length distribution of the contigs and unigenes were shown in Table 2. A total of 130,953 contigs (length ≥ 100) were assembled with the N50 of 636 nt, an average length of 352 nt, and a total nucleotides length of 46,146,957 nt. Among them, there were 109,269 contigs (83.72%) size ranging from 100 to 500 nt, 11,699 contigs (8.93%) with size varying from 501 to 1000 nt, and 9985 contigs (7.62%) with size more than 1000 nt. Thereafter, with pair-end reads, the contigs were further generated into 70,168 unigenes with a total length of 50,277,812 nt, and with an N50 of 994 nt and a mean length of 717 nt. Meanwhile, according to a sequence similarity search with known proteins or nucleotides database, a total of 70,168 consensus sequences were assigned to 32,332 clusters and 37,846 singletons. Table 2 also showed that the length of assembled unigenes were mostly ranged from 200 to 1000 nt accounted for 77.61%, and 15,713 unigenes (22.39%) with length > 1000 nt. These results indicated that the unigenes distribution followed the contigs distribution was greater among shorter assembled sequences.

**Table 1:** **Output of the transcriptome sequencing for the Radish**.

**Feature**	**Statistic**
Total Raw Reads	57,031,638
Total Clean Reads	51,169,168
Total Clean Nucleotides (nt)	4,605,225,120
Q20 percentage	98.29%
N percentage	0.01%
GC percentage	47.10%

**Table 2:** **Length distribution of assembled contigs and unigenes**.

**Nucleotides length (nt)**	**Contigs**	**Contigs percent (%)**	**Unigenes**	**Unigenes percent (%)**
100–200	74216	56.67	0	0.00
201–300	19874	15.18	17228	24.55
301–400	9788	7.47	10018	14.28
401–500	5391	4.12	7006	9.98
501–600	3596	2.75	5586	7.96
601–700	2668	2.04	4540	6.47
701–800	2109	1.61	3847	5.48
801–900	1778	1.36	3306	4.71
901–1000	1548	1.18	2924	4.17
1001–1100	1297	0.99	2360	3.36
1101–1200	1098	0.84	2117	3.02
1201–1300	935	0.71	1685	2.40
1301–1400	868	0.66	1540	2.19
1401–1500	762	0.58	1319	1.88
1501–1600	659	0.50	1088	1.55
1601–1700	589	0.45	970	1.38
1701–1800	523	0.40	771	1.10
1801–1900	416	0.32	663	0.94
1901–2000	353	0.27	503	0.72
2001–2100	308	0.24	442	0.63
2101–2200	270	0.21	389	0.55
2201–2300	217	0.17	279	0.40
2301–2400	197	0.15	235	0.33
2401–2500	176	0.13	226	0.32
2501–2600	158	0.12	154	0.22
2601–2700	124	0.09	128	0.18
2701–2800	123	0.09	104	0.15
2801–2900	112	0.09	92	0.13
2901–3000	93	0.07	82	0.12
≥3000	707	0.54	566	0.81
Total number	130953		70168	
Total nucleotides length (nt)	46,146,957		50,277,812	
Mean length (nt)	352		717	
N50 (nt)	636		994	
Total consensus sequences			70168	
Distinct clusters			32332	
Distinct singletons			37846	

Recently, several transcriptome studies of radish leaf and root had been reported (Wang et al., 2013; Zhang et al., 2013; Wu et al., 2015). Wu et al. (2015) reported that 68,086 unigenes with an average length of 576 bp and an N50 of 773 bp was generated from radish leaves by Trinity assembly. Wang et al. (2013) showed that 73,084 unigenes with a mean length of 763 nt and an N50 of 1095 nt were obtained from radish root transcriptome. In this study, the comparison analysis showed that the number and N50 sizes of the assembled unigenes were larger than those from the previous leaf transcriptome, while smaller than those from the root transcriptome (Wang et al., 2013; Zhang et al., 2013; Wu et al., 2015). These results implied that the quality of sequencing data was high enough to ensure the accuracy of the sequence assembly.

### Functional annotation of the assembled unigenes

**Figure 1:**
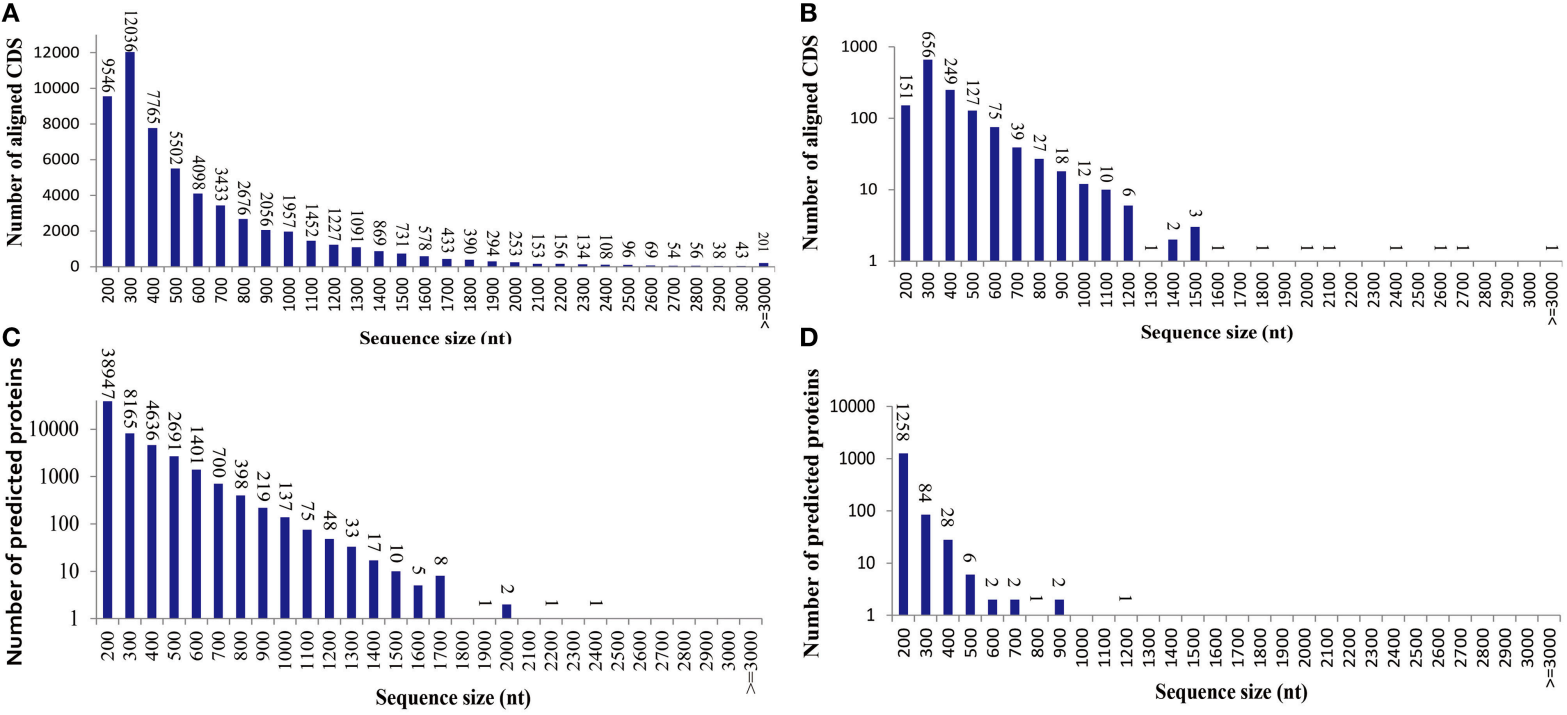
**The length distribution of the coding sequence (CDS) and predicted proteins by BLASTx and ESTScan software from the unigenes. (A)** Aligned CDS by BLASTx. **(B)** Aligned CDS by ESTScan. **(C)** Predicted proteins by BLASTx. **(D)** Predicted proteins by ESTScan.

To learn an overview information of unigene sequences from radish root transcriptome, a homology based method was adopted in unigenes annotation. The unigene sequences were performed against public protein and nucleotide databases (Nr, Swiss-Pot, KEGG, COG and Nt) using BLAST algorithm (*E* ≤ 10^−5^) to search for sequence similarity. The results of functional annotation were shown in Table 3. Out of the 70,168 unigenes, 63,991 (91.20%) unigenes were matched with the public databases. The percent of annotated unigenes was similar to previously studies in radish (92.09%) by Wang et al. (2013), suggesting that the assembled unigenes have the relatively conserved functions and this project has captured the majority of the radish transcriptome. In addition, the present study 57,495 and 1384 CDS were obtained by Blast and ESTScan alignment, respectively. However, the remaining of 6177 unigene sequences, which may represent novel genes specifically expressed in radish taproot or could be attributed to other technical or biological biases such as assembly parameters, were found to be without a homologous hit in the public databases. The length distribution of CDS and predicted proteins by BLASTx and ESTScan software were shown in Figure 1.

**Table 3:** **Summary of annotations of ‘NAU-YH’ unigenes in public databases**.

**Public database**	**No. of unigene hit**	**Percent (%)**
NR	57,303	81.67
NT	62,073	88.46
Swiss-prot	36,854	52.52
KEGG	30,971	44.14
COG	17,587	25.06
GO	51,981	74.08
ALL	63,991	91.20

**Figure 2:**
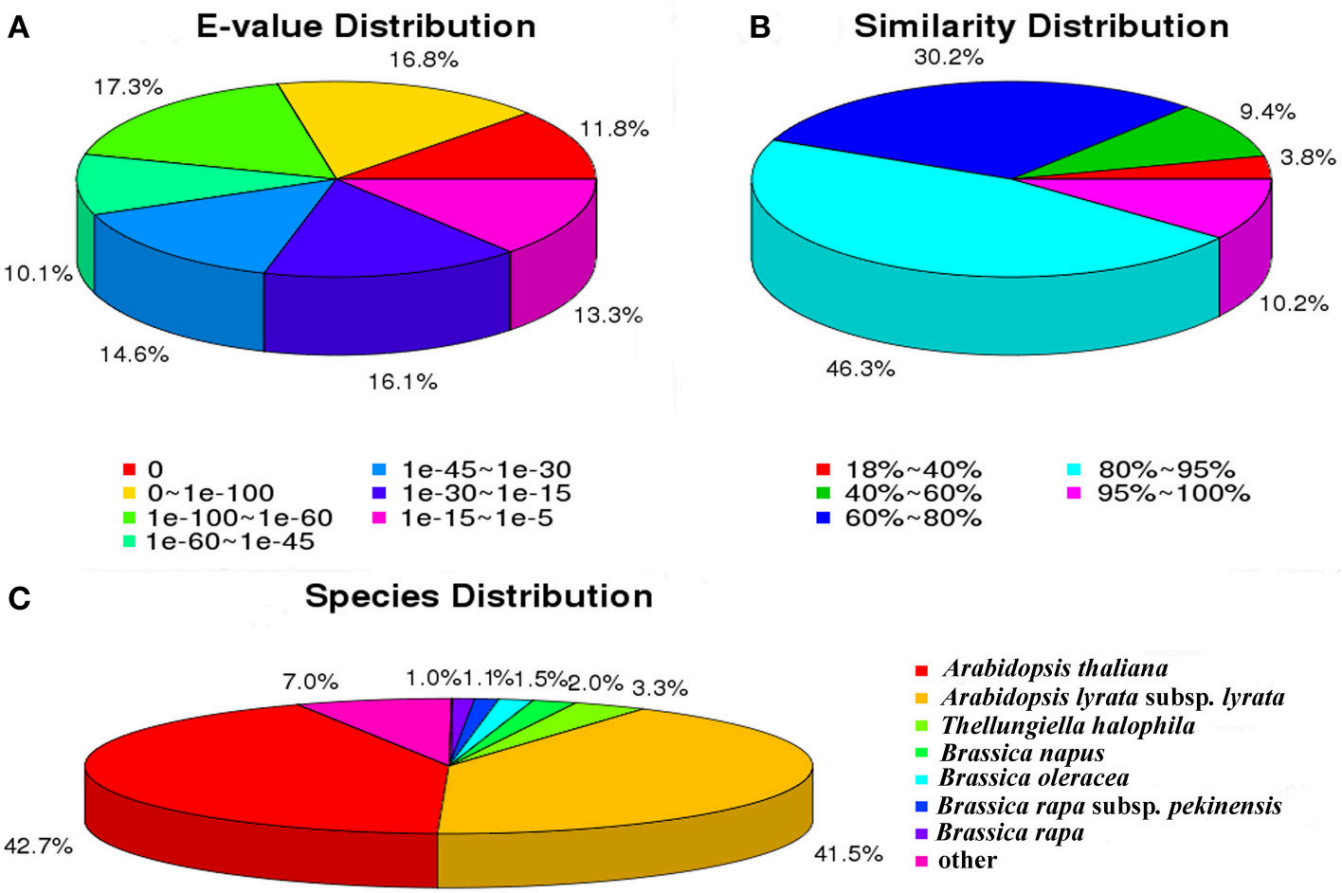
**Characteristics of sequence homology of radish root with BLAST against NCBI non-redundant (NR) database. (A)** *E*-value distribution of BLAST hits for matched unigene sequences, using an *E*-value cutoff of 10^−5^. **(B)** Similarity distribution of top BLAST hits for each unigene. **(C)** Species distribution of the top BLAST hits.

For the Nr annotations, we further analyzed the *E*-value, similarity and species distribution of the top hits in the Nr database, and the results was listed in Figure 2. The *E*-value distribution of the top hits in the Nr database indicated that 55.97% of the mapped sequences have significant homology (*E* < 1.0e^−45^), whereas the other 44.03% of the moderate homology sequences varied from 1.0e^−5^ to 1.0e^−45^ (Figure 2A). The similarity distribution displayed 56.55% of the query sequences with a similarity >80%, while 43.45% of the hits have a similarity ranging from 18 to 80% (Figure 2B). For the species distribution, we found that the majority of annotated sequences were similar to *Arabidopsis thaliana* (42.7%) and *A. lyrata* subsp. *Lyrata* (41.5%), followed by *Thellungiella halophile* (3.28%), *Brassica napus* (1.96%), *B. oleracea* (1.53%), *B. rapa* subsp. *Pekinensis* (1.06%), *B. rapa* (1.01%), and others (6.96%; Figure 2C). The BLASTx species distribution showed a bias toward *A. thaliana* and *A. lyrata subsp*. *Lyrata*, as well as five species with BLAST hits belonged to the Brassicaceae family, implying that the sequences of the radish transcripts obtained in the present study were assembled and annotated properly.

### Functional classification by GO and COG

**Figure 3:**
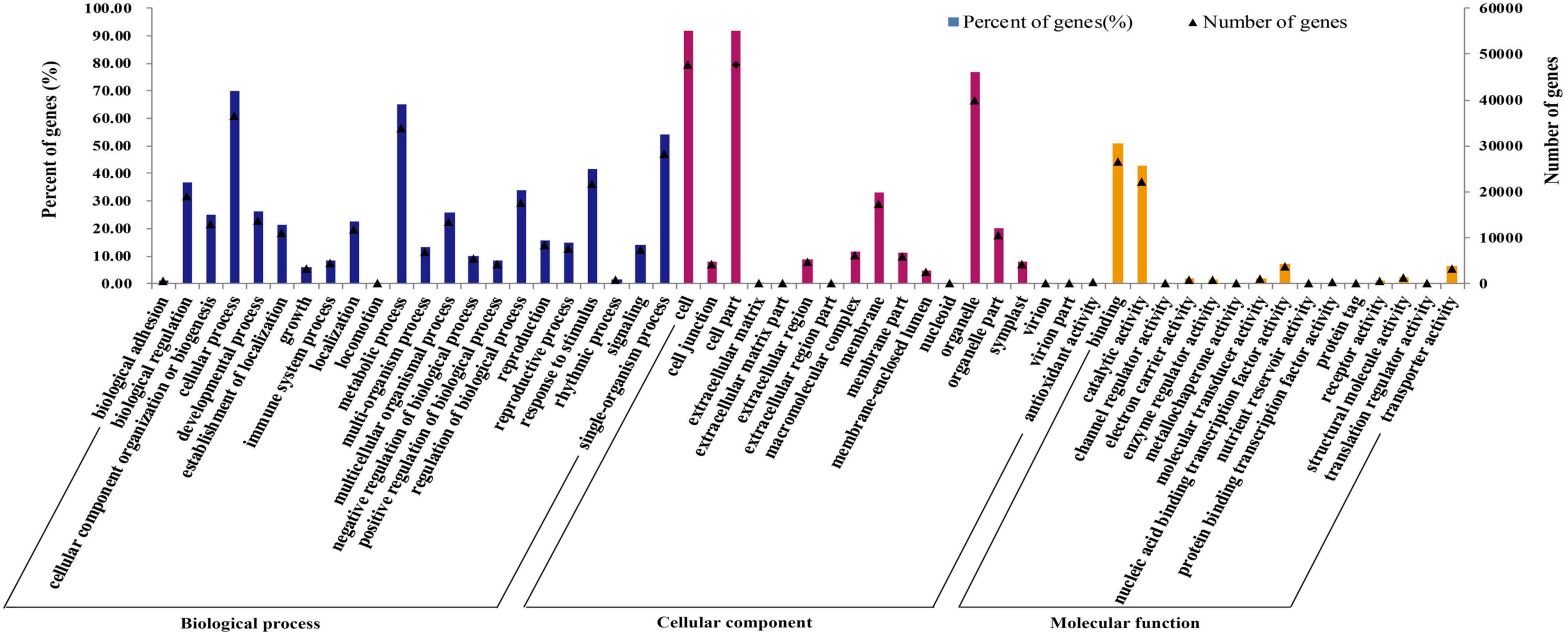
**Frequencies and mean expression levels of transcripts matching GO terms**. The percentage of transcripts matching GO terms is showing for each category as different color bars and the normalized mean expression levels of transcripts matching each of these GO terms are shown as black triangles.

Gene ontology (GO) was applied to comprehensively describe the properties of genes and their products in our transcriptome library of radish, which is an international standardized gene functional classification system. Based on the sequence similarity, 51,981 unigenes (74.08%) were categorized into 55 functional groups and summarized into three main GO categories including molecular function, cellular component and biology process (Table 3; Figure 3). Under the biological process category, “cellular process” (70.08%), and “metabolic process” (65.19%), were represented the most abundant of the category, suggesting that some important metabolic activities occured in root, these results were similar to previously reported study of *de novo* transcriptome analysis in radish (Wang et al., 2013) and sweet potato (Wang et al., 2010). Under the cellular component category, “cell” (91.67%) and “cell part” (91.67%) terms were prominently represented. For the category of molecular function, “binding” (51.01%) and “catalytic activity” (42.68%) were the most dominant represented terms. Moreover, only a few genes were assigned with “virion” (0.01%), “virion part” (0.01%), “protein tag” (0.01%), and “translation regulator activity” (0.01%) GO terms.

**Figure 4:**
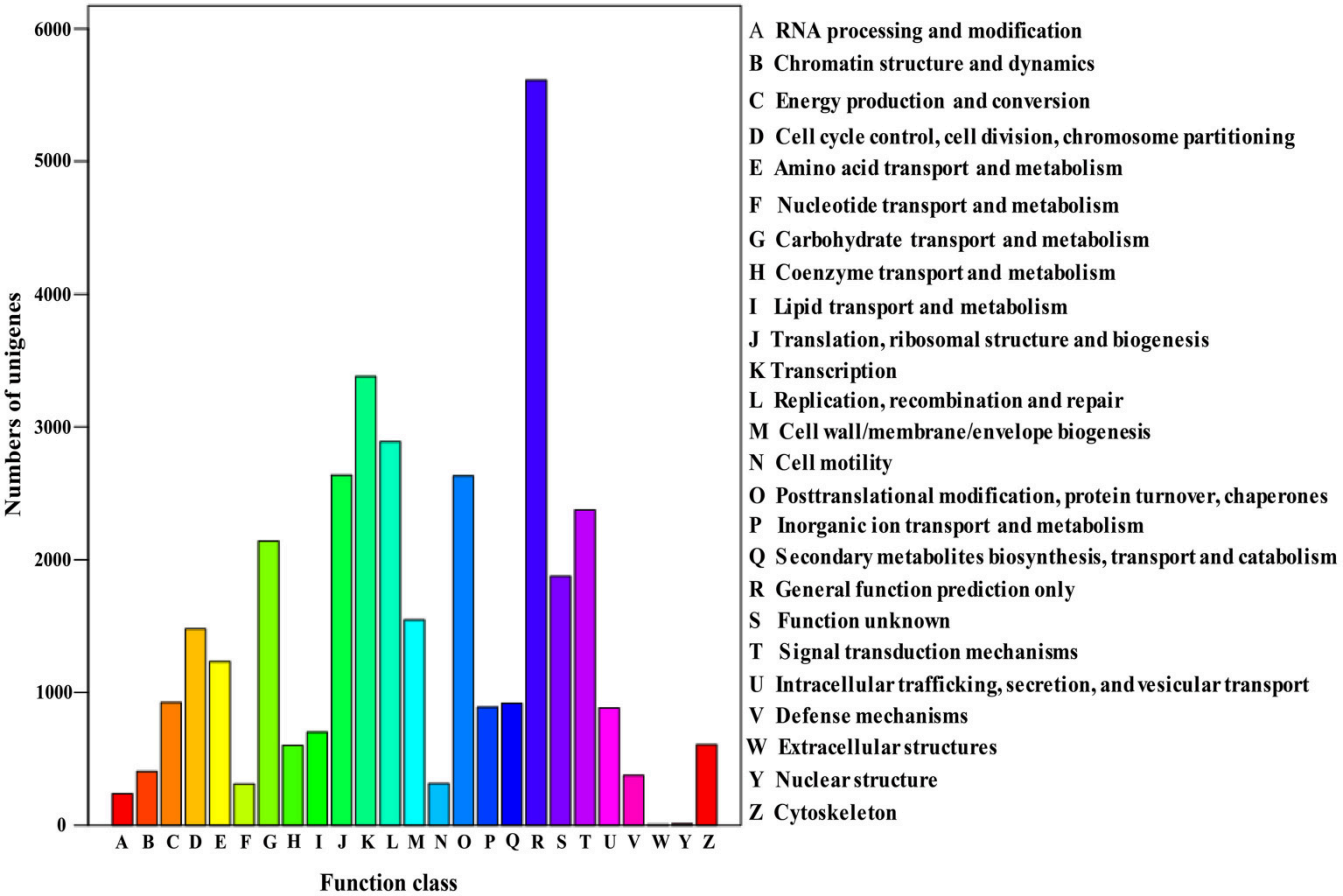
**Histogram presentation of clusters of orthologous groups (COG) classification**.

The Cluster of Orthologous Groups (COG) is a database where orthologous gene products are classified. Every protein in COG is assumed to evolve from an ancestor protein, and the whole database is built on coding proteins with complete genome as well as system evolution relationships of bacteria, algae, and eukaryotic creatures (Wang et al., 2010; Hyun et al., 2012). In this study, in order to predict and classify possible functions, the assembled unigenes were aligned to COG database. In total, 17,587 of 70,168 (25.06%) unigenes were assigned to the COG classifications (Table 3), which were grouped into 25 function categories (Figure 4). Due to some unigenes were annotated with multiple COG functions, altogether 34,972 functional annotations were generated. Among them, the five largest group included “General function prediction” (5610, 31.90%), “Transcription” (3380, 19.22%), “Replication, recombination, and repair” (2889, 16.43%), “Translation, ribosomal structure, and biogenesis” (2636, 14.99%) and “Posttranslational modification, protein turnover, chaperones” (2361, 14.96%). Conversely, five smallest groups included “Extracellular structures” (4, 0.02%), “Nuclear structure” (11, 0.06%), “RNA processing and modification” (238, 1.35%), “Nucleotide transport and metabolism” (309, 1.76%) and “Cell motility” (312, 1.77%).

### Functional classification by KEGG

**Figure 5:**
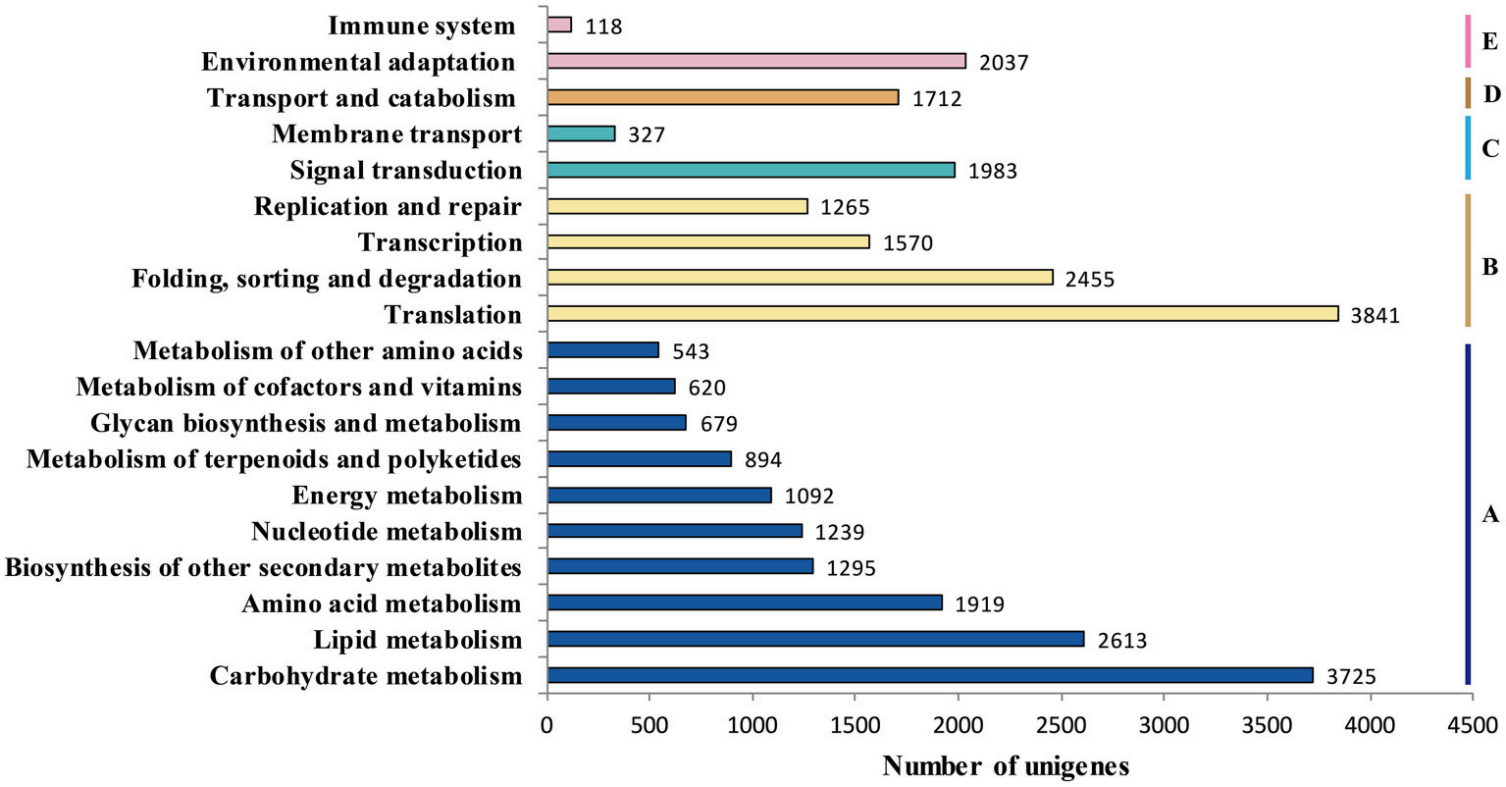
**Pathway assignment based on KEGG. (A)** metabolism; **(B)** genetic information processing; **(C)** environmental information processing; **(D)** cellular processes; **(E)** environmental adaptation.

Genes within the same pathway usually cooperate with each other to perform their biological function, suggesting that pathway-based analysis can help further understanding of the genes' functions (Wenping et al., 2011). The Kyoto Encyclopedia of Genes and Genomes (KEGG) is a protein database that is able to analyze gene product during metabolism process and related gene function in the cellular processes. Therefore, to identify the biological pathways being active in the taproot of radish, the assembled unigenes were mapped to KEGG protein database. Based on the sequence similarity, 30,971 unigenes could be assigned to 126 pathways (Table S2), which were grouped into five groups (Figure 5). These groups most represented by unigenes were metabolism (14,619 unigenes) and genetic information processing (9131 unigenes), followed by organismal systems (2155 unigenes), cellular processes (1712 unigenes) and environmental information processing (770 unigenes). In metabolism pathway (Figure 5), 14,619 unigenes were divided into 10 sub-categories, of which most representation by unigenes were carbohydrate metabolism (3725 unigenes), lipid metabolism (2613 unigenes), amino acid metabolism (1919 unigenes), biosynthesis of other secondary metabolites (1295 unigenes), and nucleotide metabolism (1239 unigenes). Taken together, the putative KEGG pathways identified in the present study elucidated specific responses and functions involved in the molecular processes of radish taproot development, and provided a resource for further investigating specific pathways in radish including the sucrose metabolism pathway.

### Analysis of sucrose metabolism pathway genes using radish unigenes

**Figure 6:**
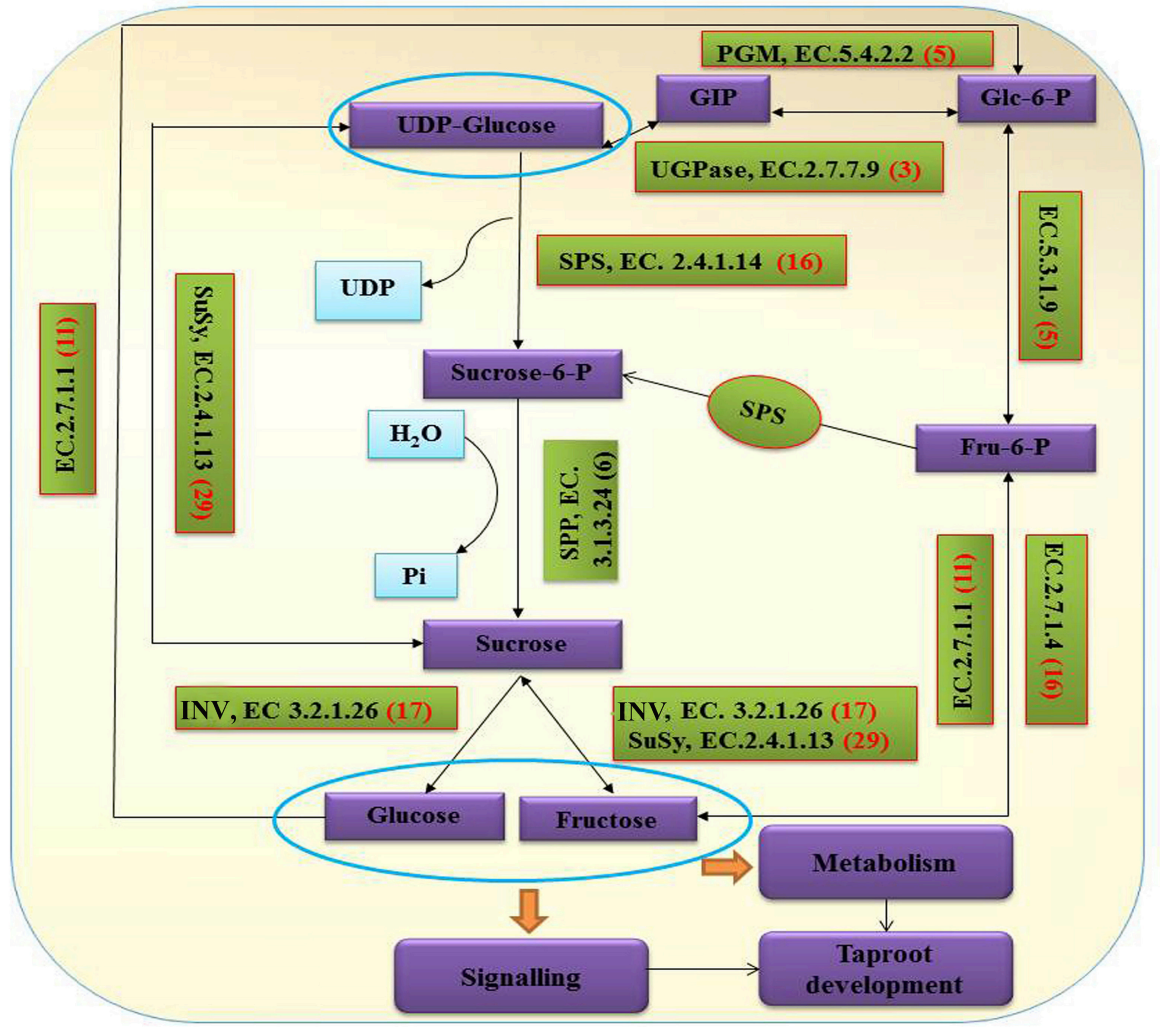
**Assembled radish unigenes that may be involved in the sucrose metabolism pathway**. The numbers in brackets following each gene name indicate the number of transcriptome unigenes annotated to that gene.

Sucrose is the major product of photosynthesis, and it is the main substrate for sink strength, and used to sustain cell metabolism and growth (Tognetti et al., 2013; Ruan, 2014). In radish, the storage root is a major sink, which begins to thicken early in development (Usuda et al., 1999). To date, the main pathway of sucrose metabolism has been well-known in higher plant (Ruan, 2012, 2014; Zhang et al., 2015). In our annotated radish taproot transcriptome dataset, a total of 103 transcripts encoding eight well-known enzymes involved in the main sucrose metabolism pathway were identified by KEEG protein database (Figure 6). Transcript IDs from the sucrose metabolism pathway were listed in Table S3A. The sucrose biosynthesis in cytosol has been proposed by two key enzymes: SPS (EC 2.4.1.14, 16 transcripts) and Sucrose-phosphate phosphatase (SPP; EC 3.1.3.24, no annotated transcripts available by KEEG protein database) (Ruan, 2012; Zhang et al., 2015), that is, Glucose (Glc) use hexokinase (EC 2.7.1.1, 11 transcripts) as substrates to generate Glc-6-phosphate (Glc-6-P), which can be converted to fructose-6-phosphate (Fru-6-P) by Glc-6-P isomerase (EC.5.3.1.9, five transcripts). Following this reaction, SPS uses Fru-6-P and UDP-Glc as substrates to produce Sucrose-phosphate (Sucrose-6-P), which is then converted to sucrose by sucrose-phosphatase or sucrose-6-phosphate phosphohydrolase (SPP; EC 3.1.3.24, six transcripts by Nr annotation and BLASTx manual) (Table S3A; Table 4). Furthermore, evidence shows that the import and accumulation of sucrose in storage roots might involve its inversion into hexose sugars for use in diverse ways by invertase and sucrose synthase (Ruan, 2014). In this study, many transcripts encoding critical functional enzyme involved in two possible sucrose degradation pathways were also discovered in our transcriptome. One is the conversion of sucrose to glucose and fructose by invertase or beta-fructofuranosidase (EC 3.2.1.26, 17 transcripts). Another is the conversion of sucrose to UDP-Glucose and fructose by SuSy (EC 2.4.1.13, 29 transcripts; Table S3A; Table 4; Figure 6). In addition, as shown in Table 4, SuSy, INV, and SPS were encoded by the high numbers of transcripts, implying that these enzymes are the major source of sucrose metabolism activity in radish (Ren and Zhang, 2013). Moreover, significant numbers of transcripts for fructokinase metabolism may represent its property of the taproot having a sweet taste (Zhang et al., 2015).

**Table 4:** **Classification analysis of unigenes coding for 8 protein families in sucrose metabolism in radish taproot transcriptomes**.

**Family**	**KEGG annotation [international enzyme name]**	**Unigene no.^b^**	**NAU-YH_raw fragments (no.)^c^**	**NAU-YH_RPKM^d^**
1^a^	sucrose-phosphate synthase [EC:2.4.1.14]	16	6254	195.175
2^a^	sucrose synthase [EC:2.4.1.13]	29	24824	1072.2468
3^a^	invertase (beta-fructofuranosidase) [EC:3.2.1.26]	17	1609	134.0041
4^a^	hexokinase [EC:2.7.1.1]	11	2290	102.1245
5^a^	UTP–glucose-1-phosphate uridylyltransferase [EC:2.7.7.9]	3	8622	278.7609
6^a^	phosphoglucomutase [EC:5.4.2.2]	5	4006	141.776
7^a^	glucose-6-phosphate isomerase [EC:5.3.1.9]	5	2838	112.6653
8^a^	fructokinase [EC:2.7.1.4]	17	13035	531.1143
9	sucrose-phosphatase or sucrose-6-phosphate phosphohydrolase (EC 3.1.3.24)	6	1381	67.7794

a Gene families from 1 to 8 were all identified from “starch and sucrose metabolism” category according to the KEGG protein database;

b The numbers under “Unigene no.” column represent the total number of unigenes in each enzyme family identified in the radish taproot transcriptome;

c The numbers under the “NAU-YH_raw fragments (no.)” column represent the raw fragments in each enzyme family identified in the radish taproot transcriptome;

d *The numbers under “RPKM” columns represent the total values of unigene RPKM in each enzyme families identified in the radish taproot transcriptome*.

To investigate which transcripts were unique involved in the main sucrose metabolism pathway in annotated ‘NAU-YH’ taproot transcriptome dataset, the transcripts encoding eight well-known enzymes involved in the main sucrose metabolism pathway by KEEG protein database were annotated from ‘NAU-RG’ taproot transcriptome dataset available in our lab [SRX316199 and http://www.ncbi.nlm.nih.gov/sra/] (Wang et al., 2013). A total of 127 transcripts were annotated in ‘NAU-RG’ taproot transcriptome dataset (Table S3B). Among of these, SuSy, INV, and SPS were also encoded by the higher numbers of transcripts. In addition, the ‘NAU-YH’ transcripts encoding eight well-known enzymes in the main sucrose metabolism pathway by KEEG protein database (Table S3A) were compared to the transcripts of ‘NAU-RG’ (Table S3B) by using local BLASTN with an *E*-value cutoff of 1e^−20^ (Table S3C). As a result, all transcripts (Table S3A) in ‘NAU-YH’ showed significant identity to the transcripts of ‘NAU-RG’ (Table S3C). These results indicated that the transcripts encoding enzymes in the main sucrose metabolism pathway were similar in these two radish genotypes.

### Validation and expression analysis of genes involved in sucrose metabolism

To assess the quality of the assembly and annotation data from radish taproot transcriptome sequencing, full-length cDNA sequences of two key genes from sucrose metabolism process were isolated by T-A cloning with the Sanger method and compared with the assembled sequences. The length of *RsSPS1* and *RsSuSy*1 genes were 3265 and 2163 bp, respectively (Table 5). Overall, the assembled unigenes covered 92.75% (*RsSPS1*) and 98.28% (*RsSuSy*1) of the corresponding full-length genes. Additionally, *RsSPS1*, and *RsSuSy*1 genes were predicted to contain the complete ORF, and the ORF pairwise identity of *RsSPS1* and *RsSuSy*1were 96.99 and 98.75%, respectively (Table 5). These results validated that the NGS-based RNA-seq procedures was reliable (Table 5).

**Table 5:** **Sequence analyses of the two putative radish genes involved in sucrose metabolism process**.

**Gene**	**Full-length cDNA (bp)**	**Unigene No**.	**Coverage (%)**	**ORF similarity (%)**
*RsSPS1*	3265	9	92.75	96.99
*RsSuSy*1	2163	13	98.28	98.75

**Figure 7:**
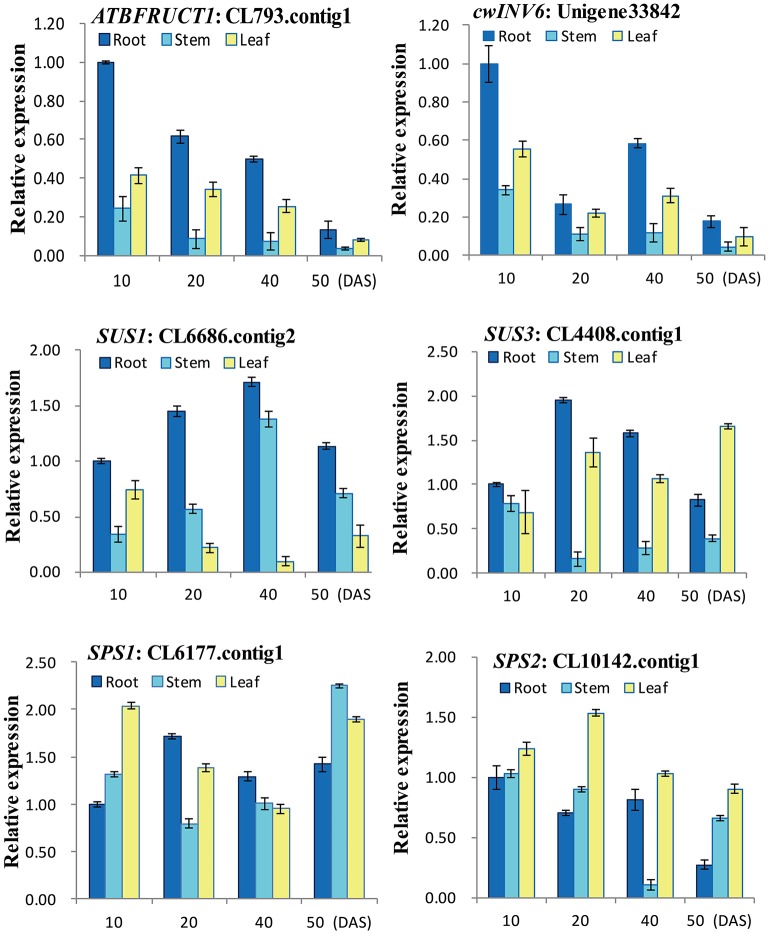
**RT-qPCR analysis of six genes involved in sucrose metabolism with different tissues and stages in radish**.

To experimentally confirm that the unigenes obtained from sequencing and computational analysis were indeed expressed, six unigenes related to sucrose metabolism pathway were chosen for RT-qPCR analysis (Figure 7). The RT-qPCR analysis showed that all the genes exhibited different expression and regulation during radish taproot thickening. The expression profiles of *ATBFRUCT1* and *cwINV6* were similar in radish different tissues and different development stages. And they were highly expressed in the root and leaf, especially in root organ of pre-cortex splitting stage (10 DAS). *SUS1* had higher expression profiles in root organ during the different taproot thickening stages. The highest expression level of *SUS1* was observed in root organ of expanding stage (40 DAS). *SUS3* exhibited higher expression in root and leaf from cortex splitting stage (20 DAS) to mature stage (50 DAS), the highest expression level in root organ at cortex splitting stage, whereas higher expression was observed in leaf at mature stage. The results were consistent with previous studies (Usuda et al., 1999; Rouhier and Usuda, 2001), suggesting they may be involved in radish taproot formation. *SPS1* was highly expressed in stem at mature stage. In contrast, the expression levels of *SPS2* in leaves were higher in roots and stems during the taproot formation, and the highest expression level of *SPS2* was observed at cortex splitting stage.

## Conclusion

In summary, a cDNA library was sequenced using NGS-based Illumina sequencing platform. From ~51 million clean reads, a total of 70,168 unigene with a total length of 50.28 Mb, an average length of 717 bp and a N50 of 994 bp were obtained. In total, 63,991 (about 91.20% of the assembled unigenes) unigenes were successfully annotated to five public databases including NR, GO, COG, KEGG, and Nt. GO term analysis revealed that the majority of these unigenes were predominately involved in basic physiological and metabolic processes, catalytic, binding and cellular process. Furthermore, a total of 103 unigenes encoding eight enzymes in the sucrose metabolism related pathways were identified. These results provided an solid foundation for identifying taproot thickening-related critical genes and would facilitate further dissecting molecular mechanisms underlying taproot formation in radish.

## Accession code

The RNA SEQ raw data of this study have been deposited in NCBI Sequence Read Archive (SRA, http://www.ncbi.nlm.nih.gov/Traces/sra) with accession number: SRX707630.

## Author contributions

YR and LW designed the experiments. YR, XL and WY performed the radish cultivation and sample collection. YR, WR, ZW and XY performed the experiments. YR wrote the manuscript draft. LW, ZX, KB and XL edited and revised the manuscript. All authors read and approved the final manuscript.

### Conflict of interest statement

The authors declare that the research was conducted in the absence of any commercial or financial relationships that could be construed as a potential conflict of interest.

## References

[B1] Angeloni F. Wagemaker C. A. Jetten M. S. Op den Camp H. J. Janssen megens E. M. Francoijs K. J.. (2011). *De novo* transcriptome characterization and development of genomic tools for *Scabiosa columbaria* L. using next-generation sequencing techniques. Mol. Ecol. Resour. 11, 662–674. 10.1111/j.1755-0998.2011.02990.x 21676196

[B2] Babb V. M. Haigler C. H. (2001). Sucrose phosphate synthase activity rises in correlation with high-rate cellulose synthesis in three heterotrophic systems. Plant Physiol. 127, 1234–1242. 10.1104/pp.010424 11706202 PMC129291

[B3] Chaturvedi P. (2008). Inhibitory response of *Raphanus sativus* on lipid peroxidation in albino rats. Evid. Based Complement. Alternat. Med. 5, 55–59. 10.1093/ecam/nel077 18317549 PMC2249733

[B4] Cheng W. H. Chourey P. S. (1999). Genetic evidence that invertase-mediated release of hexoses is critical for appropriate carbon partitioning and normal seed development in maize. Theor. Appl. Genet. 98, 485–495. 10.1007/s001220051096

[B5] Conesa A. Götz S. García Gómez J. M. Terol J. Talón M. Robles M. (2005). Blast2GO: a universal tool for annotation, visualization and analysis in functional genomics research. Bioinformatics 21, 3674–3676. 10.1093/bioinformatics/bti610 16081474

[B6] Curtis I. S. (2003). The noble radish: past, present and future. Trends Plant Sci. 8, 305–307. 10.1016/S1360-1385(03)00127-4 12878009

[B7] Farrar J. Pollock C. Gallagher J. (2000). Sucrose and the integration of metabolism in vascular plants. Plant Sci. 154, 1–11. 10.1016/S0168-9452(99)00260-5 10725553

[B8] Grabherr M. G. Haas B. J. Yassour M. Levin J. Z. Thompson D. A. Amit I.. (2011). Full-length transcriptome assembly from RNA-Seq data without a reference genome. Nat. Biotechnol. 29, 644–652. 10.1038/nbt.1883 21572440 PMC3571712

[B9] Gutiérrez R. M. Perez R. L. (2004). *Raphanus sativus* (Radish): their chemistry and biology. Scientific World Journal 4, 811–837. 10.1100/tsw.2004.131 15452648 PMC5956417

[B10] Hyun T. K. Rim Y. Jang H. J. Kim C. H. Park J. Kumar R.. (2012). *De novo* transcriptome sequencing of Momordica cochinchinensis to identify genes involved in the carotenoid biosynthesis. Plant Mol. Biol. 79, 413–427. 10.1007/s11103-012-9919-9 22580955

[B11] Iseli C. Jongeneel C. V. Bucher P. (1999). ESTScan: a program for detecting, evaluating, and reconstructing potential coding regions in EST sequences. Proc. Int. Conf. Intell. Syst. Mol. Biol. 138–148. 10786296

[B12] Johnston J. S. Pepper A. E. Hall A. E. Chen Z. J. Hodnett G. Drabek J.. (2005). Evolution of genome size in *Brassicaceae*. Ann. Bot. 95, 229–235. 10.1093/aob/mci016 15596470 PMC1950721

[B13] Kitashiba H. Li F. Hirakawa H. Kawanabe T. Zou Z. Hasegawa Y.. (2014). Draft sequences of the radish (*Raphanus sativus* L.) genome. DNA Res. 21, 481–490. 10.1093/dnares/dsu014 24848699 PMC4195494

[B14] Li X. Q. Zhang D. (2003). Gene expression activity and pathway selection for sucrose metabolism in developing storage root of sweet potato. Plant Cell Physiol. 44, 630–636. 10.1093/pcp/pcg080 12826628

[B15] Ren X. Zhang J. (2013). Research progresses on the key enzymes involved in sucrose metabolism in maize. Carbohydr. Res. 368, 29–34. 10.1016/j.carres.2012.10.016 23318271

[B16] Rouhier H. Usuda H. (2001). Spatial and temporal distribution of sucrose synthase in the radish hypocotyl in relation to thickening growth. Plant Cell Physiol. 42, 583–593. 10.1093/pcp/pce071 11427677

[B17] Ruan Y. L. (2012). Signaling role of sucrose metabolism in development. Mol. Plant 5, 763–765. 10.1093/mp/sss046 22532605

[B18] Ruan Y. L. (2014). Sucrose metabolism: gateway to diverse carbon use and sugar signaling. Annu. Rev. Plant Biol. 65, 33–67. 10.1146/annurev-arplant-050213-040251 24579990

[B19] Stokes M. E. Chattopadhyay A. Wilkins O. Nambara E. Campbell M. M. (2013). Interplay between sucrose and folate modulates auxin signaling in *Arabidopsis*. Plant Physiol. 162, 1552–1565. 10.1104/pp.113.215095 23690535 PMC3707552

[B20] Tognetti J. A. Pontis H. G. Martínez Noël G. M. (2013). Sucrose signaling in plants: a world yet to be explored. Plant Signal. Behav. 8:e23316. 10.4161/psb.23316 23333971 PMC3676498

[B21] Usuda H. Demura T. Shimogawara K. Fukuda H. (1999). Development of sink capacity of the “storage root” in a radish cultivar with a high ratio of “storage root” to shoot. Plant Cell Physiol. 40, 369–377. 10.1093/oxfordjournals.pcp.a029552

[B22] Wang F. Sanz A. Brenner M. L. Smith A. (1993). Sucrose synthase, starch accumulation, and tomato fruit sink strength. Plant Physiol. 101, 321–327. 12231688 10.1104/pp.101.1.321 PMC158679

[B23] Wang L. He Q. (2005). Chinese Radish. Beijing: Scientific and Technical Documents Publishing House.

[B24] Wang S. Wang X. He Q. Liu X. Xu W. Li L.. (2012). Transcriptome analysis of the roots at early and late seedling stages using Illumina paired-end sequencing and development of EST-SSR markers in radish. Plant Cell Rep. 31, 1437–1447. 10.1007/s00299-012-1259-3 22476438

[B25] Wang W. Gong Y. Liu L. Wang Y. Jing Z. Huang D. (2007). Changes of sugar content and sucrose metabolizing enzyme activities during fleshy taproot development in radish (*Raphanus sativus* L.). Acta Hortic. Sin. 34, 1313–1316. 10.3321/j.issn:0513-353x.2007.05.043

[B26] Wang Y. Pan Y. Liu Z. Zhu X. Zhai L. Xu L.. (2013). *De novo* transcriptome sequencing of radish (*Raphanus sativus* L.) and analysis of major genes involved in glucosinolate metabolism. BMC Genomics 14:836. 10.1186/1471-2164-14-836 24279309 PMC4046679

[B27] Wang Z. Fang B. Chen J. Zhang X. Luo Z. Huang L.. (2010). *De novo* assembly and characterization of root transcriptome using Illumina paired-end sequencing and development of cSSR markers in sweetpotato (*Ipomoea batatas*). BMC Genomics 11:726. 10.1186/1471-2164-11-726 21182800 PMC3016421

[B28] Ward J. A. Ponnala L. Weber C. A. (2012). Strategies for transcriptome analysis in nonmodel plants. Am. J. Bot. 99, 267–276. 10.3732/ajb.1100334 22301897

[B29] Weber H. Borisjuk L. Wobus U. (1997). Sugar import and metabolism during seed development. Trends Plant Sci. 2, 169–174. 10.1016/S1360-1385(97)85222-3

[B30] Wenping H. Yuan Z. Jie S. Lijun Z. Zhezhi W. (2011). *De novo* transcriptome sequencing in *Salvia miltiorrhiza* to identify genes involved in the biosynthesis of active ingredients. Genomics 98, 272–279. 10.1016/j.ygeno.2011.03.012 21473906

[B31] Wu G. Zhang L. Yin Y. Wu J. Yu L. Zhou Y.. (2015). Sequencing, *de novo* assembly and comparative analysis of *Raphanus sativus* transcriptome. Front. Plant Sci. 6:198. 10.3389/fpls.2015.00198 26029219 PMC4428447

[B32] Xiong Y. McCormack M. Li L. Hall Q. Xiang C. Sheen J. (2013). Glucose-TOR signalling reprograms the transcriptome and activates meristems. Nature 496, 181–186. 10.1038/nature12030 23542588 PMC4140196

[B33] Xu Y. Zhu X. Gong Y. Xu L. Wang Y. Liu L.. (2012). Evaluation of reference genes for gene expression studies in radish (*Raphanus sativus* L.) using quantitative real-time PCR. Biochem. Biophys. Res. Commun. 3, 398–403. 10.1016/j.bbrc.2012.06.119 22771808

[B34] Yang L. Xu M. Koo Y. He J. Poethig R. S. (2013). Sugar promotes vegetative phase change in *Arabidopsis thaliana* by repressing the expression of MIR156A and MIR156C. Elife 2:e00260. 10.7554/eLife.00260 23538384 PMC3608266

[B35] Ye J. Fang L. Zheng H. Zhang Y. Chen J. Zhang Z.. (2006). WEGO: a web tool for plotting GO annotations. Nucleic Acids Res. 34, W293–W297. 10.1093/nar/gkl031 16845012 PMC1538768

[B36] Zhang L. Jia H. Yin Y. Wu G. Xia H. Wang X.. (2013). Transcriptome analysis of leaf tissue of *Raphanus sativus* by RNA sequencing. PLoS ONE 8:e80350. 10.1371/journal.pone.0080350 24265813 PMC3827192

[B37] Zhang L. Lin Q. Feng Y. Fan X. Zou F. Yuan D. Y.. (2015). Transcriptomic identification and expression of starch and sucrose metabolism genes in the seeds of Chinese Chestnut (*Castanea mollissima*). J. Agric. Food Chem. 63, 929–942. 10.1021/jf505247d 25537355

